# Cells shed from tumours show reduced clonogenicity, resistance to apoptosis, and in vivo tumorigenicity

**DOI:** 10.1038/sj.bjc.6690760

**Published:** 1999-11

**Authors:** M A Swartz, C A Kristensen, R J Melder, S Roberge, E Calautti, D Fukumura, R K Jain

**Affiliations:** Department of Radiation Oncology, andCutaneous Biology Research Center, Massachusetts General Hospital and Harvard Medical School, Boston, MA 02114, USA

**Keywords:** tumour cell shedding, clonogenicity, tumour perfusion, experimental metastasis, tumorigenicity, metastatic potential

## Abstract

The goal of this study was to compare growth characteristics of cells shed from a tumour with the native tumour cells. The human colon adenocarcinoma LS174T and its highly metastatic subline LS LiM 6 were grown as tissue-isolated tumours in nude mice and perfused to collect shed cells. The tumours were then excised and prepared into single-cell suspensions. Clonogenicity in 0.3–0.9% agarose, apoptotic fraction, and in vivo tumorigenicity were determined for each population. In both tumour lines, shed cells were less clonogenic, more apoptotic and less tumorigenic than cells isolated directly from their native tissue. These findings suggest that shed cells have a low metastatic potential compared to native tumour cells, most likely because they represent an apoptotic population. © 1999 Cancer Research Campaign


*British Journal of Cancer* (1999) **81**(5), 756–759
© 1999 Cancer Research Campaign
Article no. bjoc.1999.0760

Short Communication

### Cells shed from tumours show reduced clonogenicity, resistance to apoptosis, and in vivo tumorigenicity

**MA Swartz[1]*, CA Kristensen[1]*, RJ Melder[1], S Roberge[1], E Calautti[2], D Fukumura[1] and RK Jain[1]**

[1]Department of Radiation Oncology and [2]Cutaneous Biology Research Center, Massachusetts General Hospital and Harvard Medical School, Boston, MA 02114, USA

**Summary** The goal of this study was to compare growth characteristics of cells shed from a tumour with the native tumour cells. The human colon adenocarcinoma LS174T and its highly metastatic subline LS LiM 6 were grown as tissue-isolated tumours in nude mice and perfused to collect shed cells. The tumours were then excised and prepared into single-cell suspensions. Clonogenicity in 0.3–0.9% agarose, apoptotic fraction, and in vivo tumorigenicity were determined for each population. In both tumour lines, shed cells were less clonogenic, more apoptotic and less tumorigenic than cells isolated directly from their native tissue. These findings suggest that shed cells have a low metastatic potential compared to native tumour cells, most likely because they represent an apoptotic population. © 1999 Cancer Research Campaign

**Keywords:** tumour cell shedding; clonogenicity; tumour perfusion; experimental metastasis; tumorigenicity; metastatic potential

Although millions of cells are shed daily from a tumour, the formation of metastases is a rare event. The seminal observations of Butler and Gullino (1975) of tumour cell shedding from a tissue-isolated mammary carcinoma, of Liotta et al (1974, 1976) of shedding from a fibrosarcoma and of Fidler (1973) of melanoma cells injected intravenously helped to establish the concept of metastasis as a highly inefficient process. In this process, specific cell phenotypes are selected for at each step in order to eventually form a distant metastatic colony (Weiss, 1992; Aznavoorian et al, 1993; Zetter, 1993; Fidler and Ellis, 1994; Chambers and Matrisian, 1997).

The goal of this study was to investigate whether tumour cell shedding is a selective step in the metastatic cascade. To this end, we compared colony-forming potential and apoptosis of cells shed from a tumour with cells isolated directly from their host tumour. The LS174T tumour line along with its highly metastatic variant LSLiM6 (Bresalier et al, 1991) were grown as tissue-isolated tumours in the ovarian fat pad of nude mice (Kristjansen et al, 1994). This preparation permits collection of all cells shed by a tumour. Tumours were perfused at different flow rates, and then digested into single-cell suspensions to obtain two different cell populations. Shed cells were compared with their native tumour cell population using in vitro clonogenicity in increasing agarose concentrations (0.3–0.9%), since clonogenicity (particularly in hard agarose) has been repeatedly correlated with metastatic potential and prognosis (Li et al, 1989; Nomura et al, 1989; Zhang et al, 1991). The LS174T cell groups were also implanted into mouse dorsal skinfold chambers (Leunig et al, 1992) to compare the relative tumorigenicity of shed and native tumour cells. Finally, the apoptotic fractions of each cell population were compared. Our results demonstrate that perfusion rate does not affect shedding rate or clonogenicity of shed cells. Furthermore, the shed cells are highly apoptotic and have reduced clonogenicity and tumorigenicity.

### MATERIALS AND METHODS

### Animals and tumours

For all experiments, 8- to 10-week-old NCr/Sed-*nu/nu* female athymic mice bred in our animal facility were used. All protocols were approved by the Massachusetts General Hospital Subcommittee of Research and Animal Care. Before each procedure, the mice were anaesthetized with a solution of ketamine (100 mg $kg^{-1}$ body weight, Parke-Davis, Morris Plains, NJ, USA) and xylazine (10 mg $kg^{-1}$, Miles, Shawnee Mission, KS, USA). The human colon adenocarcinoma line LS174T (ATCC #CL 188, Rockville, MD, USA) and its highly metastatic subline LS LiM 6 (kind gift of Dr Robert Bresalier, University of California, San Francisco, CA, USA) were serially transplanted to the flanks of donor mice to maintain stocks of donor tissue. The tissue-isolated tumours, which are connected with the systemic circulation by only a single artery and vein, were prepared as originally described by Kristjansen et al (1994) and experiments performed when the tumours reached a size of 8–12 mm in diameter. Because of the limitations imposed by the small numbers of shed cells ($10^4$–$10^5$ per experiment), each variable (clonogenicity, apoptotic fraction, tumorigenicity and cell shedding rate) was examined in independent experiments.

### Tumour perfusion

The cannulation of aorta and vena cava and the ligation of vessels surrounding the tumour artery and vein, as well as the perfusion setup, has previously been described in detail (Kristjansen et al,

*Received 6 January 1999*
*Revised 22 April 1999*
*Accepted 13 May 1999*

*Correspondence to:* RK Jain

*These two authors contributed equally to this work.

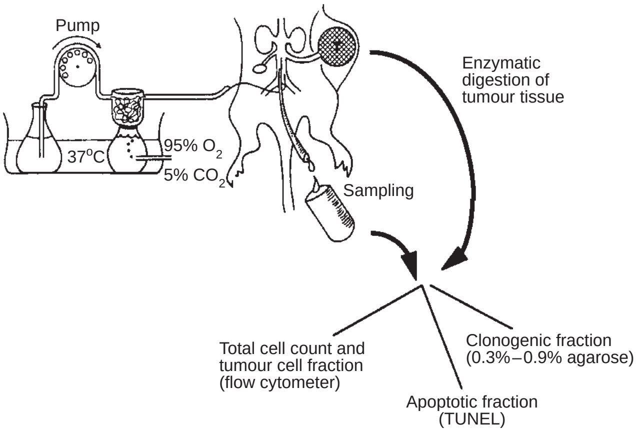

**Figure 1** Schematic of the perfused, tissue-isolated tumour setup to collect shed cells. The abdominal aorta and vena cava are cannulated as indicated and ligated below the level of the iliac truncus. All vascular branches except those draining and feeding the tissue-isolated tumour are ligated. *T* denotes the tissue-isolated tumour in a sealed parafilm bag. Two-minute samples were collected and either counted for total number of live cells and then plated for determination of clonogenicity or sorted in a flow cytometer for determination of tumour cell fraction of the total cell population. Modified from Kristjansen et al (1994)

1994, 1996) and is depicted in Figure 1. In the present experiments, the tumours were perfused with oxygenated horse serum (Sigma Chemicals, St Louis, MO, USA) at 37°C. Sterility of the serum was maintained by filtering the perfusate through a sterile syringe filter (25 mm, 0.2 μm, Corning Glass Works, Corning, NY, USA) before flowing into the tumour through γ-irradiated PE-10 polyethylene tubing. For the studies comparing the effects of perfusion rate on cell shedding rate, the initial perfusate flow rate was set at 65 μl $min^{-1}$, which was at the low end of the physiological tumour blood flow range of 30–322 μl $min^{-1}$ $g^{-1}$ (Kristjansen et al, 1994). The outlet perfusate was collected in 2-min samples for 20 min, wherefore the flow rate was increased to 90, 110 and, if possible, 120 μl $min^{-1}$ (with 2-min sampling for 20 min at each level). For all other experiments, perfusion rate was fixed at 100 μl $min^{-1}$ and collected for 2 h. At the end of each perfusion, the tumour was excised and weighed.

### Cell isolation from solid tumours

Tissue-isolated tumours were minced and rinsed with Dulbecco's modified Eagle's medium (DMEM) (Sigma) with 10% fetal calf serum (FCS; Sigma). For every 1 g of tissue, 2 ml of collagenase type III solution (1 mg $ml^{-1}$ in RPMI-1640, 109 units $mg^{-1}$; Cooper Biomedical, Malvern, PA, USA) was added to the tissue. The tissue suspension was incubated at room temperature for 4 h, filtered through a 70-μm nylon cell strainer (Becton-Dickinson, Franklin Lakes, NJ, USA) and the resulting cell suspension was washed twice in medium and counted in a haemocytometer. For assessment of colony-forming ability, cells were diluted to concentrations approximately equal to that of the average shed cell count.

### Cell counts

The first set of experiments evaluated the effect of varying perfusion rate utilizing LS174T tumours. First, the volume of each 2-min perfusate sample was measured by weight. Every fifth sample (a total of three from each flow rate) was set aside for determination of tumour cell count, while the rest were analysed for colony formation. The total live cell count was measured in every other sample using trypan blue exclusion.

Because the LS174T tumour line is of human origin, tumour cells could be marked with a generic HLA antibody and counted in a flow cytometer. The shed cells were compared with the minced tumour cells. Cultured mouse endothelial cells (ATCC) were used as a negative control and human lymphocytes as a positive control. Each sample was labelled with fluoroscein isothiocyanate (FITC)-conjugated mouse anti-human HLA Class I-ABC antibody (Biosource Int., Camarillo, CA, USA) and fixed in 3% paraformaldehyde. Flow cytometry was performed with a FACScan (Becton-Dickinson).

To evaluate the effects of perfusion rate on colony formation, each sample was washed and incubated in one well of a 12-well plate containing 3 ml DMEM and 30 000 irradiated (60 Gy, 1 fraction) LS174T 'feeder' cells (Puck and Marcus, 1955). After 21 days, the plates were stained with 1% 3-(4,5-dimethylthiazal-2-yl)-2,5-diphenyltetrazolium bromide (MTT; Sigma) in DMEM (1.5 mg $ml^{-1}$, 2 ml per well) and examined for colony size. Colonies were identified as spherical cell clusters which were greater than 100 μm in diameter. Medium with 30 000 irradiated LS174T cells served as a negative control.

### Clonogenicity in varying agarose concentrations

For determination of in vitro clonogenicity, each cell population from the LS174T or LS LiM 6 tumours was perfused and prepared as described earlier and incubated in concentrations of 1000, 5000, 10 000 or 20 000 cells per well in 6 well plates. Cell plating number was evaluated in pilot experiments using both LS174T and LS LiM 6 cells taken directly from culture. Each plate contained 1.5 ml of 1.2% agarose gel in DMEM at the bottom. Above this was a cell layer containing 1.5 ml of 0.3%, 0.6%, or 0.9% agarose in DMEM with 10% FCS. To the top of each well was added 1 ml DMEM with 10% FCS, which was changed every fifth day. The cells were allowed to grow in a 37°C humidified incubator. After 3 weeks, colonies were counted according to their size distributions using a light microscope.

### Determination of apoptotic fraction

Cytospin preparations (2 000 cells each) were made from each cell population, and from some LS174T tumours, cryostat sections were made as a control to verify that the digestion method was not killing the native apoptotic cells (which would result in a lower measured apoptotic fraction). Each slide was evaluated for apoptosis using an Apoptag Fluorescein Apoptosis Kit (Oncor Inc, Gaithersburg, MD, USA). Cells were fixed and solubilized in 70% ethanol for 4 h. Manufacturer's instructions were then followed, and 0.1% propidium iodide was used as a counterstain. Slides were observed under a fluorescence microscope (Zeiss, Oberkochen, Germany) using a 32× objective and on each slide, six regions or enough to evaluate approximately 100 cells were used. In each region, a cell count was first determined (i.e. propidium iodide-stained nuclei) and then the apoptotic-positive fraction was determined using a fluorescein filter. For the tumour sections, an average cell density was determined and the apoptotic fraction evaluated by direct counting of ten regions.

© 1999 Cancer Research Campaign

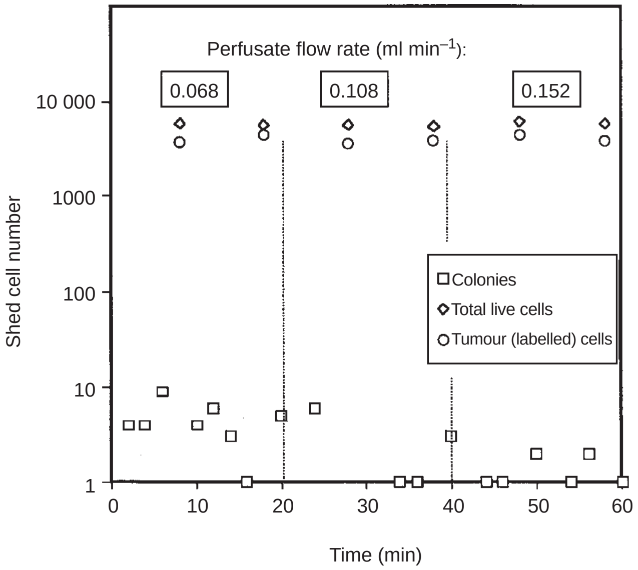

**Figure 2** Representative shed cell counts from a perfused tissue-isolated LS174T tumour. Each vertical line represents a change in perfusion rate

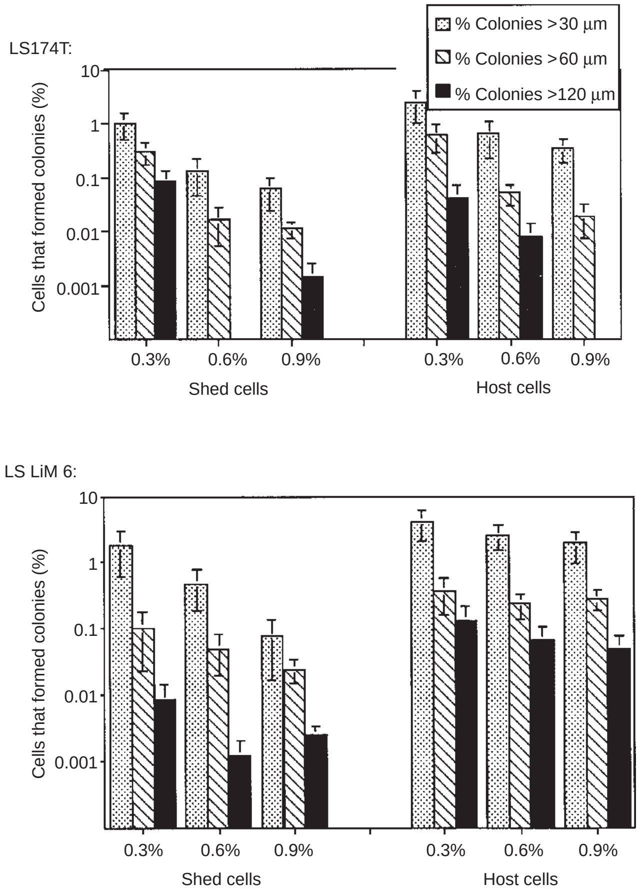

**Figure 3** Mean clonogenicity of shed cells compared to their host tumour cells in different agarose concentrations. Shed cells were significantly less clonogenic than host tumour cells in 0.6% and 0.9% agarose in both tumour lines ($P < 0.05$). LS174T cells were significantly less clonogenic than their more metastatic subline cells LS LiM 6 at agarose concentrations of 0.6% and 0.9% ($P < 0.05$). Error bars indicate standard deviations

### Tumorigenicity

To assess relative tumorigenicity, six LS174T tumours were perfused and a single-cell suspension was prepared from the host tumour as before. Cells from each population were washed twice with DMEM, spun down into a concentrated pellet and seeded into dorsal skinfold chambers as described previously (Leunig et al, 1992). Approximately $2 \times 10^5$ minced tumour cells and $3 \times 10^5$ shed tumour cells were used for each chamber, based on approximations of tumour cell fractions from each population (determined by flow cytometry) and previous experience with tumour cell numbers required to produce a tumour in a dorsal skinfold chamber (Leunig et al, 1992). Fifty percent more shed cells were seeded into the chamber than minced cells in order to ensure that the results were based solely on differences in average cell phenotype.

### RESULTS

### Tumour cell shedding rates

The perfused LS174T and LS LiM 6 tumours shed an average (± s.d.) of $2.6 \pm 0.9 \times 10^5$ and $9.8 \pm 0.5 \times 10^4$ tumour cells $h^{-1}$ $g^{-1}$ tumour respectively. This corresponds to 6.2 and 2.3 million cells $day^{-1}$ $g^{-1}$. The cell fractions staining positive for HLA were 65 ± 5% and 56 ± 14% for native and shed cells respectively (cultured cells stained 82 ± 2% positive). Cells were consistently shed as single cells; less than 0.1% were found to be in multicellular clusters. Cell isolation from solid tumour tissue also resulted in approximate single-cell suspensions; only 0.8 ± 0.4% of cell units were clusters ranging in size from 2 to 10 cells. Therefore, this discrepancy was neglected in evaluating clonogenicity and single cell suspensions were assumed (Gerweck et al, 1994).

### Perfusion rate does not affect cell shedding rate

Figure 2 shows typical cell populations shed as a function of time from a single representative experiment. Changes in perfusion rate are indicated by vertical dotted lines. Average clonogenicity on plastic of native LS174T cells (tumour digest) was 2.7 ± 1.4%, while that of the shed cells was 0.16 ± 0.07% (cultured LS174T cells showed an average clonogenicity of 4.5 ± 1.0%). No correlation was found between the cell shedding rate and perfusion rate as well as between their clonogenicity and flow rate.

### Shed cells have reduced clonogenicity

Clonogenicity of the cell populations is shown in Figure 3. The native LS LiM 6 cells showed higher clonogenicity in 0.6–0.9% agarose concentrations than the corresponding groups of the LS174T tumours ($P < 0.01$ for all colony size groups using a Student's *t*-test), and native tumour cells were consistently more clonogenic than their shed counterparts (e.g. in 0.3% agarose, $P < 0.05$ for both tumour lines). These differences were most notable at 0.6% and 0.9% agarose concentrations, particularly in the LS LiM 6 groups ($P < 0.01$ for LS LiM 6 and $P < 0.05$ for LS174T).

### Shed cells are apoptotic

In both tumour types, shed cells showed greater apoptotic fractions than the native cell populations. For the LS174T tumours,

© 1999 Cancer Research Campaign

20 ± 11% of native cells (verified by the frozen section stainings, which showed apoptotic indices between 10 and 40%) and 53 ± 12% of shed cells were apoptotic. For LS LiM 6 tumours, 9 ± 3% of native cells and 59 ± 8% of shed cells were apoptotic. Differences in apoptotic fraction between cells shed from the LS174T tumour and those from the LS LiM 6 tumour were not significant. However, differences between native populations were significant ($P < 0.05$ using a Student's *t*-test). Furthermore, for each tumour line, the shed and native cells were significantly different ($P < 0.005$ for LS174T and $P < 0.0005$ for LS LiM 6).

### Shed cells have reduced tumorigenicity

Shed cells showed reduced tumorigenicity when implanted into dorsal skinfold chambers. Every chamber implanted with minced cells (6/6) showed angiogenic activity and tumour formation (1–4 mm diam) at 3 weeks, while none of the six shed cell groups formed tumours and only one showed signs of angiogenesis.

### DISCUSSION

Metastasis is known to be an inefficient process. The events of metastasis – detachment, intravasation, survival within the bloodstream, adhesion to the vessel wall, migration and proliferation – are each selective processes which require specific cell phenotypes. The observations of Butler and Gullino (1975) and Liotta et al (1974, 1976), although seminal, did not give insight into the phenotype of the shed cells as well as the significance of shedding rates. The perfused tissue-isolated tumour model enabled us to study the initial steps in the metastatic process and to characterize the shed cells relative to the overall native tumour cell population. In particular, in vitro clonogenicity, apoptotic fraction, and in vivo tumorigenicity were examined. These traits have been strongly linked to metastatic potential. For example, more highly metastatic tumour lines are more clonogenic in 0.9% agarose (Li et al, 1989; Zhang et al, 1991) and show an increased resistance to apoptosis (Glinsky and Glinsky, 1996; Glinsky, 1997). These findings are consistent with our observed differences between native tumour cells of the LS174T and its highly metastatic variant, LS LiM 6. The total numbers of tumour cells shed from the LS174T and LS LiM 6 tumours corroborate previously published data on cell shedding rate (Butler and Gullino, 1975). However, these shed cells showed decreased proliferative capacity in vitro and in vivo, suggesting poor metastatic capacity compared with the average cell population from the native tumour. TUNEL stainings of tissue sections showed apoptotic indices directly comparable to those of cells isolated by enzymatic digestion indicating that there was no selection of apoptosis-resistant cells after enzymatic treatment. The observation that most of these shed cells are apoptotic – 48% compared with the average native cell apoptotic fraction of 20% in LS174T tumours, and 59% compared with the native fraction of 9% in the more highly metastatic variant LS LiM 6 – strengthens the hypothesis that cell shedding is a natural process of eliminating ineffective tumour cells and not a selective step in the metastatic cascade. With this hypothesis, we would expect more highly metastatic tumours to have lower apoptotic rates (Glinsky and Glinsky, 1996; Glinsky, 1997) and therefore shed fewer cells, which is consistent with the tumour lines examined here. Our finding also suggests that the low metastasis-forming potential of cancer cells is not only due to their inability to survive in the circulation, adhere, and extravasate at the secondary site, but also due to the fact that most of the cells are already in the process of dying when they exit the tumour.

### ACKNOWLEDGEMENTS

The authors are grateful to Drs Leo Gerweck and Tanweer Zaidi for technical advice, Ms Julia Kahn for technical assistance and Dr Robert Bresalier for providing LS LiM 6 cells. This work was supported by an Outstanding Investigator Grant (R35-CA-56591) from the National Cancer Institute (RKJ) and the Danish Medical Research Council (CAK). CAK is a postdoctoral fellow of the Michaelsen Foundation, Denmark. DF is a Whitaker Fellow.

#### REFERENCES

Aznavoorian S, Murphy AN, Stetler-Stevenson WG and Liotta LA (1993) Molecular aspects of tumor cell invasion and metastasis. *Cancer* **71**: 1368–1383

Bresalier RS, Niv Y, Byrd JC, Duh Q-Y, Toribara NW, Rockwell RW, Dahiya R and Kim YS (1991) Mucin production by human colonic carcinoma cells correlates with their metastatic potential in animal models of colon cancer metastasis. *J Clin Invest* **87**: 1037–1045

Butler TP and Gullino PM (1975) Quantitation of cell shedding into efferent blood of mammary adenocarcinoma. *Cancer Res* **35**: 512–516

Chambers AF and Matrisian LM (1997) Changing views of the role of matrix metalloproteinases in metastasis. *J Natl Cancer Inst* **89**: 1260–1270

Fidler IJ (1973) The relationship of embolic homogeneity, number, size and viability to the incidence of experimental metastasis. *Eur J Cancer* **9**: 223–227

Fidler IJ and Ellis LM (1994) The implications of angiogenesis for the biology and therapy of cancer metastasis. *Cell* **79**: 185–188

Gerweck LE, Dullea R, Zaidi ST, Budach W and Hartford A (1994) Influence of experimental factors on intrinsic radiosensitivity assays at low doses of radiation: cell multiplicity. *Rad Res* **138**: 361–366

Glinsky GV (1997) Apoptosis in metastatic cancer cells. *Crit Rev Oncol Hematol* **25**: 175–186

Glinsky GV and Glinsky VV (1996) Apoptosis and metastasis: a superior resistance of metastatic cancer cells to programmed cell death. *Cancer Lett* **101**: 43–51

Kristjansen PEG, Roberge S, Lee I and Jain RK (1994) Tissue-isolated human tumor xenografts in athymic nude mice. *Microvasc Res* **48**: 389–402

Kristjansen PEG, Brown TJ, Shipley LA and Jain RK (1996) Intratumour pharmacokinetics, flow resistance, and metabolism during gemcitabine infusion in *ex vivo* perfused human small cell lung cancer. *Clin Cancer Res* **2**: 359–367

Leunig M, Yuan F, Menger DM, Boucher Y, Goetz AF, Messmer K and Jain RK (1992) Angiogenesis, microvascular architecture, microhemodynamics, and interstitial fluid pressure during early growth of human adenocarcinoma LS174T in SCID mice. *Cancer Res* **52**: 6553–6560

Li L, Price JE, Fan D, Zhang R, Bucana CD and Fidler IJ (1989) Correlation of growth capacity of human tumor cells in hard agarose with their in vivo proliferative capacity at specific metastatic sites. *J Natl Cancer Inst* **81**: 1406–1412

Liotta LA, Kleinerman J and Saidel GM (1974) Quantitative relationships of intravascular tumor cells, tumor vessels, and pulmonary metastases following tumor implantation. *Cancer Res* **34**: 997–1004

Liotta LA, Kleinerman J and Saidel GM (1976) The significance of hematogenous tumor cell clumps in the metastatic process. *Cancer Res* **36**: 889–894

Nomura Y, Tashiro H and Hisamatsu K (1989) In vitro clonogenic growth and metastatic potential of human operable breast cancer. *Cancer Res* **49**: 5288–5293

Puck TT and Marcus PI (1955) A rapid method for viable cell titration and clone production with HeLa cells in tissue culture: the use of x-irradiated cells to supply conditioning factors. *Proc Natl Acad Sci USA* **41**: 432–437

Weiss L (1992) Biomechanical interactions of cancer cells with the microvasculature during hematogenous metastasis. *Cancer Met Rev* **11**: 227–235

Zetter BR (1993) Adhesion molecules in tumor metastasis. *Sem Cancer Res* **4**: 219–229

Zhang RD, Price JE, Schackert G, Itoh K and Fidler IJ (1991) Malignant potential of cells isolated from lymph node or brain metastases of melanoma patients and implications for prognosis. *Cancer Res* **51**: 2029–2035

© 1999 Cancer Research Campaign 

